# Supplementary material for: WordCommentsAnalyzer: A windows software tool for qualitative research
Source: F1000Res. 2018 Sep 4;7:536. Originally published 2018 May 3. [Version 2] doi: 10.12688/f1000research.14819.2 (PMC6137411; doi:10.12688/f1000research.14819.2)
Supplement: Supplementary file 3 [file f1000research-7-17614-s0002.tgz › b2a79496-4ada-47c4-9682-a99ce867836d.zip!/Eight Iranian Health Journals/Journal of Knowledge and Health_17.docx]

© Journal of Knowledge and Health. This work is licensed under CC BY 4.0 (https://creativecommons.org/licenses/by/4.0/).

Volume & Issue: دوره 9، شماره 1: 1393

Title: تأثیر مصرف مکمل سیلی‌مارین و تمرین استقامتی بر ميزان مالون‌دی‌آلدئید پلاسماي مردان غیر فعال

http://knh.shmu.ac.ir/index.php/site/article/view/382

مقدمه: هدف از تحقیق حاضر بررسی تأثیر تمرینات استقامتی و مصرف مکمل سیلی‌مارین بر شاخص مالون‌دی‌آلدئید (MDA) مردان غیر فعال می‌باشد. مواد و روش‌ها: تحقیق حاضر از نوع نیمه تجربی بوده که آزمودنی‌های آن 19 نفر و از بین کلیه دانشجویان مرد غیرفعال دانشگاه صنعتی شاهرود می‌باشد. پس از کسب رضایت‌نامه، آزمودنی‌ها به‌صورت تصادفی در دو گروه مداخله (11 نفر) و دارونما (8 نفر) تقسیم و مورد مطالعه قرار گرفتند. هر دو گروه به‌مدت 6 هفته، همزمان با مصرف مکمل سیلی‌مارین یا دارونما به اجرای تمرینات استقامتی پرداختند. نمونه‌های خون قبل و بعد از دوره تمرینی و مکمل‌گیری به‌منظور اندازه‌گیری شاخص پراکسیداسیون چربی از آزمودنی‌ها گرفته شد (MDA). برای تجزیه‌و‌تحلیل یافته‌های تحقیق حاضر از نرم‌افزار SPSS16 و روش تحلیل واریانس مکرر (2×2) استفاده شد. نتايج: مصرف مکمل سیلی‌مارین همراه با تمرینات استقامتی در مقایسه با گروه دارونما موجب کاهش معنی‌داري ميزان مالون دي آلدئيد پلاسما را در گروه مکمل كاهش مي‌دهد (012/0P≤). نتیجه‌گیری: تمرین استقامتی به همراه مصرف مکمل سیلی‌مارین به‌طور معني‌داري پراکسیداسیون چربی در مقایسه با گروه دارونما را كاهش مي‌دهد. لذا به‌نظر می‌رسد نوع، مدت و شدت تمرین و مقدار مصرفی مکمل سیلی‌مارین بر میزان پراکسیداسیون چربی و در نتيجه سطح مالون دي آلدئيد پلاسما مؤثر مي‌باشد.

Title: آثار استرس مزمن شناي اجباري با تعداد جلسات مختلف بر مرحله اینتر فاز و قسمت پایانی فاز 2 در آزمون فرمالین

http://knh.shmu.ac.ir/index.php/site/article/view/406

مقدمه: استرس آثار دوگانه‌ای را بر آستانه درد و رفتار افراد متعاقب درد ايفا مي‌نمايد. استرس حاد سبب بی‌دردی مي‌شود درحالی که استرس‌های مزمن در جوندگان باعث پردردی می‌شوند. آزمون فرمالین به‌عنوان مدل درد التهابی دارای سه مرحله می‌باشد که آثار استرس مزمن شنا با تعداد جلسات مختلف، بر مراحل مختلف اين آزمون، هنوز ناشناخته باقي‌مانده است. لذا هدف این مطالعه بررسي آثار استرس مکرر شنا در تعداد جلسات مختلف پس از ايجاد درد به روش تزريق فرمالين در موش صحرایی نژاد ویستار مي‌باشد. مواد و روش‌ها: در این مطالعه، از آزمون فرمالین (50 میکرولیتر فرمالین 2%) برای ارزیابی آثار استرس مزمن شنا در تعداد جلسات مختلف روی پاسخ به درد استفاده شد. حیوانات در مدت زمان 6 دقیقه در روز در گروه‌های جداگانه و به تعدادروزهای 3 یا 5 و یا10 روزه تحت استرس شنای اجباری قرار گرفتند و سپس در 24 ساعت پس از آخرین جلسه استرس شنا تزریق فرمالین به پنجه عقبی انجام شد و رفتارهای دردی مشاهده و ثبت گردید. نتایج: استرس در تعداد جلسات 3 روزه در مدت زمانی 6 دقیقه بر فاز یک، اینتر فاز و ابتدای فاز 2 بی‌تأثير بوده و فقط بر مرحله‌ی پایانی فاز 2 مؤثر است. این افزایش به‌صورت وابسته به تعداد جلسات استرس تفاوت معناداری نمي‌کند در حالی که استرس مزمن در جلسات 5 و 10 روزه هم بر مرحله‌ی پایانی فاز 2 و هم بر مرحله اینترفاز مؤثر است به‌صورتی‌که در هر دو مرحله‌ اینترفاز و پایانی فاز 2 افزایش مي‌یابد و این افزایش وابسته به افزایش زمان استرس در هر جلسه نمي‌باشد. نتیجه‌گیری: این یافته‌ها نشان می‌دهد که استرس شنای مزمن در تعداد جلسات 3، 5 و 10 روز سبب طولانی شدن رفتارهای درد شده است و افزایش تعداد جلسات بر رفتار‌های درد در مرحله اینترفاز تأثير معني‌داري داشته که مي‌تواند ناشی از اثري طولانی مدت بر مکانیسم‌های تعدیلی درد در این مرحله باشد.

Title: بررسی تأثير برنامه آموزش رایانه‌ای بر خودکارآمدی زنان باردار در سازگاری با زایمان

http://knh.shmu.ac.ir/index.php/site/article/view/407

مقدمه: میزان زایمان سزارین در ایران، حدود 3-4 برابر میزان توصیه شده سازمان جهانی بهداشت می‌باشد. آموزش مادران، نقش کلیدی در افزایش خودکارآمدی آنان در سازگاری با زايمان، کاهش ترس از زایمان طبیعی و درنتیجه کاهش تمایل آنان به انجام سزارین دارد. لذا، این مطالعه با هدف تعیین تأثير آموزش رایانه‌ای بر خودکارآمدی زایمان زنان باردار در سازگاری با زايمان انجام شده است. مواد و روش‌ها: مطالعه حاضر کارآزمایی بالینی است که برروی 150 زن باردار نخست‌زا انجام گردید. گروه مداخله برنامه آموزش رایانه‌ای را به مدت 8-6 هفته دریافت نمودند. به‌منظور تعیین خودکارآمدی، از پرسشنامه خودکارآمدی زایمان در 32-28 هفتگی و مجدداً 38-36 هفتگی استفاده گردید. برای تجزیه‌و‌تحلیل داده‌ها، از آزمون‌های آماری کای اسکوئر، من ویتنی استفاده شد. نتايج: بعد از مداخله، اختلاف آماری معناداری بين میانه و دامنه میان چارکی خودکارآمدی زایمان، در گروه مداخله (607 و20) و کنترل (394 و 16) وجود داشت (001/0>P). همچنین اختلاف آماری معناداری در میانه نمره‌های پیامد قابل انتظار (307) و خودکارآمدی قابل انتظار (301) بعد از مداخله در هر دو مرحله زایمان بین دو گروه مشاهده گردید (001/0>P). نتیجه‌گیری: نتایج تحقیق، مؤثر و کارآمد بودن برنامه آموزشی رایانه‌ای را در افزایش خودکارآمدی زنان باردار در سازگاری با لیبر تأیید می‌نماید. از اين‌رو، استفاده از آن به‌عنوان یک روش آموزشی جهت آمادگی برای زایمان پيشنهاد می‌گردد.

Title: بررسي غلظت کادمیوم و کروم در شير مادر

http://knh.shmu.ac.ir/index.php/site/article/view/354

مقدمه: تغذیه کودکان، بالاترین اولویت را در هر برنامه با هدف مراقبت‌های بهداشتی کودکان دارد و آلودگی شیر به عناصر سمی مختلف مي‌تواند آثار نامطلوبی بر سلامت کودکان بر جای گذارد. مطالعه حاضر با هدف تعیین غلظت فلزات سنگین کادمیوم و کروم در شیر مادران در شهر ورامین انجام گردید. مواد و روش‌ها: این مطالعه به‌صورت مقطعی انجام گرفت. در تحقیق حاضر، میزان کروم و کادمیوم شیر در 100 مادر مراجعه‌کننده به درمانگاه‌های انتخاب شده در شهر ورامین در 4 تا 8 هفته پس از زایمان با روش طیف سنجی جذب اتمی شعله‌ای اندازه‌گیری گردیده و مورد بررسی قرار گرفت. نتایج: میانگین (±انحراف معیار) سطح کادمیوم در شیر مادر، μg /ml9/6 ± 5 و سطح کروم معادل  μg /ml7/2 ± 3 بود. نتایج رگرسیون خطی نشان داد افرادی که محل زندگی آنها نزدیک به کارخانه یا مرکز صنعتی بوده سطح کادمیوم در شیر آنان بالاتر بوده است. همچنین، میزان سطح کروم در شیر مادر در موارد مصرف سیگار توسط همسر، مصرف برنج وارداتی، مصرف آب معدنی و زندگی نزدیک به کارخانه یا مرکز صنعتی، بالاتر بود. نتیجه‌گیری: مطالعه حاضر ارتباط وجود برخی عوامل از‌جمله وجود کارخانه یا مرکز صنعتی نزدیک محل زندگی، سیگارکشیدن همسر، نوع برنج و آب مصرفی را با سطح کادمیوم و کروم در شير مادر نشان داد. سطح کادمیوم و کروم شیر مادران در این مطالعه بالاتر از سطوح این عناصر در مقالات بررسی شده و استاندارد جهانی بود لذا به‌کارگیری راهکارهایی جهت کاهش این عناصر، جهت بهبود سلامت جامعه مناسب می‌باشد. از آنجا برخی متغیرها از‌جمله وجود کارخانه یا مرکز صنعتی نزدیک محل زندگی، سیگارکشیدن همسر، نوع برنج و آب مصرفی می‌تواننند بر میزان ورود این عناصر در شیر مادر تأثير داشته باشند، مي‌توان با به‌کارگیری تمهیداتی، عناصر مذکور در شیر مادر را كاهش داده يا حذف نمود.

Title: نقش آموزش بهداشت در کاهش عوامل خطر بیماری‌های قلبی- عروقی: یک مرور نظام‌مند

http://knh.shmu.ac.ir/index.php/site/article/view/349

مقدمه: بیماری‌های قلبی- عروقی یکی از 10 علت اصلی مرگ در دنیا و اولین علت مرگ در ایران مي‌باشند. با کنترل عوامل خطر این بیماری‌ها مي‌توان بیش از 50% ناتوانی‌های ناشی از این بیماری‌ها را کاهش داد. در این راستا در نظر داشتن رفتارهای مرتبط با سبک زندگی افراد اهمیت دارد. هدف از نگارش این مقاله، بررسی مداخلات آموزشي به‌منظور کاهش عوامل خطر بیماری‌های قلبی- عروقی براساس روش کار و چگونگی استفاده از مدل‌ها و تئوری‌های آموزش بهداشت بتوان تأثير اين مداخلات را در كاهش عوامل خطر بررسي نمود. مواد و روش‌ها: جستجوی الکترونیکی پایگاه‌های اطلاعاتی با استفاده از کلمات کلیدی فارسی و انگلیسی انجام شد. پایگاه‌های مورد بررسی عبارت بودند از بانک اطلاعاتی مقالات علوم پزشکی ایران (Iran Medex)، پایگاه اطلاعات علمی (SID)، Pubmed، Ebsco و Cochrane. جستجوی پایگاه‌های فوق‌الذکر از بهمن 1390 تا اردیبهشت 1391 انجام شد. نتایج: مداخلات در زمینه کاهش عوامل خطر بیماری‌های قلبی- عروقی به دودسته کلی مبتنی بر مدل‌ها و تئوری‌های آموزش بهداشت و بدون استفاده از مدل‌ها و تئوری‌های آموزش بهداشت تقسیم شدند. از 19 مقاله بررسی شده 9 مطالعه مبتنی بر مدل‌ها و تئوری‌های آموزش بهداشت و 10 مطالعه بدون استفاده از مدل‌ها و تئوری‌های آموزش بهداشت انجام شده بود. نتیجه‌گیری: نتایج به‌دست آمده نشان‌دهنده این موضوع هستند که آموزش‌های مبتنی بر تئوری و مدل‌های آموزش بهداشت و استفاده از برنامه‌های استاندارد شده تأثير بیشتری بر کاهش عوامل خطر بیماری‌های قلبی- عروقی دارند. زمان، مکان و طول انجام مداخلات، ویژگی‌های افراد و استفاده از روش‌های نوین آموزشی برای برخی از مخاطبان در اثر بخشی آموزش‌ها به‌منظور کاهش عوامل خطر اهمیت به‌سزايي دارند.

Title: بررسي ميزان اعتبار مارکرهاي خوني در تشخيص هويت انسان با انگشت‌نگاري DNA

http://knh.shmu.ac.ir/index.php/site/article/view/346

مقدمه: تعيين گروه‌هاي خوني اولين مرحله در تأييد يا رد کردن رابطه قرابت (خويشاوندي) بین دو فرد در تشخيص هويت است. در اين مطالعه هدف، اعتبارسنجي سيستم‌هاي گروه‌هاي خوني رايج، در تشخيص هويت افراد با روش انگشت‌نگاري DNA است. مواد و روش‌ها: در اين مطالعه از 300 فرد متعلق به 150 خانواده خون ‌گرفته شد. DNA هر فرد استخراج و انگشت‌نگاري DNA براي ده منطقه STR توسط دستگاه ABI انجام شد. هفت سيستم گروه خوني شامل: ABH، RH، MNSs، Kell، Kidd، Lutheran و P1 به‌روش اگلوتيناسيون براي هر يک از افراد مشخص شد و يافته‌ها با نتايج تعيين توالي دستگاه ABI مقايسه شد. نتايج: از 300 مورد بررسي در 256 مورد هيچ‌گونه اختلافي بين نتايج سيستم‌هاي خوني و انگشت‌نگاري DNA وجود نداشت، در 44 مورد رابطه قرابت ازطريق DNA Typing اثبات گرديد ولي ناسازگاري آنتي‌ژنيک در آنتي‌ژن‌هاي RH و MNS و ABH مشاهده گرديد، از بين سيستم‌هاي گروه خوني، سيستم‌هاي RH، MNSs و ABH به‌ترتيب با ميزان خطايي برابر 7/30%، 001/0=P، 54/20% 04/0=P و 6/17% 005/0=P ضعيف‌ترين سيستم‌ها براي تشخيص هويت شناخته شدند. نتيجه‌گيري: از بين سيستم‌هاي گروه خوني مارکرهاي RH، MNSs و ABH به‌ترتيب پرخطاترین مارکرها براي بررسي رابطه قرابت هستند و در استناد به نتايج آنها بايد حتماً از انگشت‌نگاري DNA به‌عنوان يک آزمون تأيید‌کننده استفاده نمود.

Title: تأثیر الگوی توانمندسازی خانواده محور بر دانش و کنترل متابولیکی بیماران مبتلا به دیابت نوع دو

http://knh.shmu.ac.ir/index.php/site/article/view/348

مقدمه: نقش برنامه‌هاي مناسب آموزشی در کنترل مطلوب بیماری دیابت شناخته شده است. همچنین توجه به آموزش خانواده بیماران ديابتي برای همکاری فعال در مراقبت و درمان از اهمیت ویژه‌ای برخوردار می‌باشد. هدف از انجام پژوهش حاضر تعیین تأثیر به‌کارگیری الگوی توانمندسازی خانواده محور بر دانش و کنترل متابولیکی بیماران مبتلا به دیابت نوع 2 بود. مواد و روش‌ها: در این مطالعه کارآزمایی بالینی شاهددار، 103 بیمار دیابتی پس از انجام پیش آزمون با انجام تخصیص تصادفی به‌روش طبقه‌بندی بلوکی در دو گروه آزمون و کنترل وارد شدند. براي بررسی دانش از پرسشنامه محقق ساخته استفاده نموده و شاخص HbA1C به‌عنوان کنترل متابولیکی به‌کار گرفته شد. سپس مداخله براساس الگوی توانمندسازی خانواده محور جهت گروه آزمون اجرا و پس از سه ماه مجدداً بررسي دانش به كمك پرسشنامه انجام گرفته و مقادير HbA1C نيز اندازه‌گيري شد. برای تجزیه‌و‌تحلیل داده‌ها از نرم‌افزار آماری SPSS نسخه 16 و آزمون‌های آماری کای دو، تی مستقل و تی زوجی استفاده شد. نتايج: دو گروه از نظر متغیرهای دموگرافیک با يكديگر همگن بودند. بين نمره دانش و میزان HbA1C قبل و بعد در گروه کنترل تفاوت معنادار وجود نداشت (05/0P>) اما در گروه آزمون این تفاوت معنادار بود. همچنین با آزمون تی مستقل بعد از مداخله، بین این دو گروه تفاوت معناداري در نمره دانش و میزان HbA1C مشاهده گرديد (05/0P<). نتیجه‌گیری: نتایج مطالعه حاکی از آثار مثبت آموزش مبتنی بر الگوی توانمندسازی خانواده محور در گروه مداخله در مقایسه با آموزش‌های رایج بود، لذا با‌توجه به نقش خانواده در سلامت بیماران، استفاده از این روش آموزشی به پرستاران و مسئولین مربوطه جهت کمک به کنترل متابولیکی بهینه در بیماری دیابت توصیه مي‌شود.

Title: مقايسه‌ي سطح سرمی آهن، فریتین و TIBC در کودکان 9-59 ماهه تب‌دار با كودكان تب ‌‌تشنجي

http://knh.shmu.ac.ir/index.php/site/article/view/358

مقدمه: درصد بسيار كمي از تب‌ها باعث تشنج شده و تغیرات مقادير برخي از مواد موجود در خون نيز مي‌تواند باعث تشديد و يا كاهش بروز تشنج شود. هدف از اين مطالعه مقايسه سطح سرمي آهن و فريتين در بين كودكان تب تشنجي و تب‌دار بدون تشنج مي‌باشد. مواد و روش‌ها: اين پژوهش برروي 485 بيمار 9 تا 59 ماهه تب‌دار مراجعه‌كننده به بيمارستان امام حسين (ع) شاهرود در سال 90 انجام شده است. در اين مطالعه بيماران به دو گروه مورد (كودكان تب تشنجي) و گروه شاهد (كودكان فقط تب‌دار) تقسيم شده و پس از انجام اقدامات اوليه،‌ تثبيت علائم حياتي،‌ كنترل تشنج و تب، خون وريدي از همه آنها گرفته شده و آهن سرم، ظرفيت اتصال آهن تام‌ (TIBC) و فريتين با روش استاندارد اندازه‌گيري شده است. نتايج: در اين مطالعه، 165 كودك در گروه کودکان تب تشنجی و 320 كودك در گروه شاهد قرار گرفتند. از اين بيماران، 1/49% در گروه مورد و 6/51% در گروه شاهد مذكر بودند. ميانگين سني كودكان 4/14 ± 1/32 ماه در گروه مورد و 4/15 ± 2/34 ماه در گروه شاهد بود. ميانگين زمان تب در كودكان گروه مورد 2/1 ± 2/3 روز و در گروه شاهد 2/1 ± 0/4 روز بود. ميانگين مدت زمان تشنج 7/0 ± 2/1 دقيقه و ميانگين دفعات تشنج 3/1 ±0/1 مرتبه به‌دست آمد. نتایج این پژوهش نشان داد که مقادیر آهن (003/0P<)، فریتین (008/0P<) و TIBC (001/0P<) به‌طور معناداری در گروه مورد بیشتر از شاهد بود. همچنين اين مطالعه نشان داد كه انواع درجات آنمي در گروه شاهد (7/38%) بيشتر از گروه مورد (1/29%) بوده است. نتيجه‌گيري: نتايج اين پژوهش نشان داد كه سطح المان‌هاي آهن در كودكان دچار تب و تشنج بالاتر از كودكان بدون تشنج بوده و به‌نظر مي‌رسد سطوح پايين‌تر آهن مي‌تواند نقش محافظتي در پيشگيري از تب تشنج داشته باشد.

Title: خطرپذیری مصرف سیگار در نوجوانان: ارائه مدل تعاملی نظارت والدینی و همنشینی با همسالان منحرف

http://knh.shmu.ac.ir/index.php/site/article/view/381

مقدمه: نوجوانی مرحله حساسی برای شروع رفتار پرخطر مصرف سیگار محسوب می‌شود. پژوهش حاضر با هدف بررسی نقش نظارت والدینی و همنشینی با همسالان منحرف در مصرف سیگار نوجوانان صورت گرفته است. مواد و روش‌ها: مطالعه حاضر از نوع همبستگی با استفاده از روش الگویابی معادلات ساختاری است. جامعه آماری پژوهش متشکل از کلیه نوجوانان دختر و پسر مقطع متوسطه شهر تهران بود که 1241 نفر از آنها با استفاده از روش نمونه‌گیری خوشه‌ای چندمرحله‌ای انتخاب شدند. برای گردآوری داده‌ها از مقیاس‌های خطرپذیری نوجوانان ایرانی، نظارت والدینی و همنشینی با همسالان منحرف استفاده شد. داده‌ها با استفاده از شاخص‌های آماری همبستگی t مستقل و مدل‌سازی معادلات ساختاری، مورد تجزیه‌و‌تحلیل قرار گرفتند. نتایج: نتایج پژوهش نشان داد بین پسران و دختران در خطرپذیری مصرف سیگار تفاوتی وجود ندارد، میزان نظارت والدینی در دختران و میزان همنشینی با همسالان منحرف در پسران بیشتر بود. نظارت والدینی از‌طریق همنشینی با همسالان دارای تأثير معنا‌دار بر مصرف سیگار می‌باشد و مدل واسطه‌ای ارايه شده قادر است 10% از واریانس مصرف سیگار را تبیین می‌کند. نتیجه‌گیری: توجه جدی به نقش نظارت والدینی در کاهش همنشینی با همسالان منحرف و به‌دنبال آن کاهش احتمال خطرپذیری مصرف سیگار از سوی نوجوانان از اهمیت بالایی برخوردار است.

Title: تأثير سولفید هیدروژن سدیم بر اختلالات نورولوژيكي رفتاري ناشي از ایسکمی مغزي- موضعی در موش صحرایی نر

http://knh.shmu.ac.ir/index.php/site/article/view/465

مقدمه: ايسكمي مغزي سومين علت مرگ‌و‌مير و دومين علت معلوليت‌هاي نورولوژيکي بعد از بيماري آلزايمر در جهان است. NaHS که در سیستم‌های بیولوژیک تولید سولفید هیدروژن (H2S) می‌کند، باعث کاهش آسیب پس از ايسكمي در بافت‌هاي مختلف مي‌شود. مطالعات قبلی نشان داده‌اند H2S آثار محافظتي در برابر آسيب ناشي از ايسکمي قلب ايفا مي‌كند. علاوه بر این H2S آثار محفاظتی بر سلول‌های هیپوکامپ موش و تورن‌هاي ناحيه قشر مخ دز محيط كشت دارد. لذا اين مطالعه با هدف تعيين آثار حفاظتی  NaHS بر آسيب‌هاي ناشي از ايسكمي مغزي- موضعي انجام گرفت. مواد و روش‌ها: ايسکمي مغزي - موضعي با مسدود کردن شريان مياني مغز به مدت يک ساعت و سپس برقراري مجدد جريان خون توسط روش فیلامنت به مدت 23 ساعت ايجاد شد. اثر تزريق داخل صفاقی NaHS (با دوز 1 و 5 ميلي‌گرم) در شروع ايسكمي بر اختلالات نورولوژيكي رفتاري و ادم مغزي بررسي گرديد. نتایج: تجويز داخل صفاقی NaHS با دوزهای 1 و 5 در شروع ايسکمي به‌طور معناداری (10/0±81 و 15/0± 35/78) باعث کاهش ادم مغزی نسبت‌به گروه ایسکمی شد (01/0P<). NaHS با دوزهای 1 و 5 میلی‌گرم اثر معناداري بر اختلالات نورولوژيكي حركتي نداشت (05/0P>). نتيجه‌گيري: يافته‌هاي اين تحقيق نشان داد NaHS با كاهش ادم مغزي اثر محافظتي در مدل تجربي ايسكمي مغزي-موضعي در موش صحرايي نر ايفا مي‌كند.
